# Saturating representation of loop conformational fragments in structure databanks

**DOI:** 10.1186/1472-6807-6-15

**Published:** 2006-07-04

**Authors:** Narcis Fernandez-Fuentes, András Fiser

**Affiliations:** 1 Department of Biochemistry and Seaver Foundation Center for Bioinformatics, Albert Einstein College of Medicine, 1300 Morris Park Avenue, Bronx, NY 10461, USA

## Abstract

**Background:**

Short fragments of proteins are fundamental starting points in various structure prediction applications, such as in fragment based loop modeling methods but also in various full structure build-up procedures. The applicability and performance of these approaches depend on the availability of short fragments in structure databanks.

**Results:**

We studied the representation of protein loop fragments up to 14 residues in length. All possible query fragments found in sequence databases (*Sequence Space*) were clustered and cross referenced with available structural fragments in Protein Data Bank (*Structure Space*). We found that the expansion of PDB in the last few years resulted in a dense coverage of loop conformational fragments. For each loops of length 8 in the current Sequence Space there is at least one loop in Structure Space with 50% or higher sequence identity. By correlating sequence and structure clusters of loops we found that a 50% sequence identity generally guarantees structural similarity. These percentages of coverage at 50% sequence cutoff drop to 96, 94, 68, 53, 33 and 13% for loops of length 9, 10, 11, 12, 13, and 14, respectively. There is not a single loop in the current Sequence Space at any length up to 14 residues that is not matched with a conformational segment that shares at least 20% sequence identity. This minimum observed identity is 40% for loops of 12 residues or shorter and is as high as 50% for 10 residue or shorter loops. We also assessed the impact of rapidly growing sequence databanks on the estimated number of new loop conformations and found that while the number of sequentially unique sequence segments increased about six folds during the last five years there are almost no unique conformational segments among these up to 12 residues long fragments.

**Conclusion:**

The results suggest that fragment based prediction approaches are not limited any more by the completeness of fragments in databanks but rather by the effective scoring and search algorithms to locate them. The current favorable coverage and trends observed will be further accentuated with the progress of Protein Structure Initiative that targets new protein folds and ultimately aims at providing an exhaustive coverage of the structure space.

## Background

Functional characterization of proteins is one of the most frequent problems in biology. While sequences provide valuable information, their high plasticity makes it frequently impossible to identify functionally relevant residues. For instance, it is estimated that 75% of homologous enzymes share less than 30% identical positions[1]. Meanwhile less than 30% of related protein pairs above 50% sequence identity have entirely identical EC numbers[2]. Functional characterization of a protein is usually facilitated by its three-dimensional (3D) structure[3]. These structures can be obtained by experiments, such as X-ray crystallography, NMR spectroscopy, Cryo-electron microscopy, or by computation. It has been recognized that technically complicate, time consuming and expensive 3D experimental approaches will not catch up with the millions of sequences that are emerging from high-throughput projects around the world[4]. Two major computational alternatives are available[5]. The first ones are the template based approaches (comparative modeling, threading) that have been employed in the Protein Structure Initiative (PSI)[6]. PSI efforts are expected to experimentally solve ~5000 carefully selected protein folds that could serve as seed templates for comparative modeling to provide useful three dimensional models for the rest of the -hundreds of thousands of- sequences. While the resulting comparative models will be accurate for most of the structure, some of the most critical parts of the proteins may not be modeled accurately. For instance, the overall accuracy of a comparative model for a protein that belongs to one of the superfolds[7] can be very high, because there are many high resolution structures available as templates sharing the same general fold, however the variable regions of these structures are different. The variable regions are often unique in each of these proteins, and define the function and specificity of the molecules. For these unique structural segments that are often found on the surface of the proteins and without any translational symmetry (*i.e*., loops), comparative modeling techniques cannot generally be applied; loop segments in the target may be missing in the template or structurally divergent, resulting in inaccurate parts in the model. On the other hand, short fragments of proteins play a critical role in full structure buildup approaches, too. Some of the most accurate methods available assemble full protein structures by locating short segments in the databanks and packing them together using some sort of minimization protocol such as Monte Carlo simulation[8,9]. These approaches proved to be useful to provide reasonable structures (within 4–8 A RMSD to the experimental solution) for small proteins, typically less than a 100 residues[10]. To improve the accuracy of models that are already subject to computational modeling and to extend the applicability of whole structure buildup methods to more sequences it is critical to have a good selection of short building blocks in the structure databases.

The relevance of database search methods for predicting loop structures was explored in 1994 by Fidelis et al[11]. The database search approach consists of finding a segment of mainchain that fits the two stem regions of a loop [12-20]. It was concluded that only segments of 7 residues or less (4 loop and 1+2 anchor residues) had most of their conceivable conformations present in the database of known protein structures[11]. In contrast, 8 and 9-residue segments occurred more than once in less than 70% and 40% of the cases, respectively. These numbers were obtained by extrapolation, by comparing the frequency distribution of repeat conformations in PDB, where segments were clustered if they were structurally related to one other by 1 Å RMSD or less. These types of estimates strongly depend on the criteria for selecting matching conformations. Lessel and Schomburg explored the completeness of fragments in Protein Data Bank (PDB) using a similar clustering approach. Fragments were grouped if the distance between first and last Cα atoms was shorter than 1.6 Å and the RMSD, considering Cα atoms only, was smaller than 0.8 Å. Lessel and Schomburg's confirmed the conclusions of Fidelis et al[11], but with their slightly stricter criteria only short fragments of three and four residues long were well sampled in PDB despite the three years older and larger database investigated[21]. The situation is made even worse by the requirement for an overlap of at least one residue between the database fragment and the anchor core regions, which means that modeling of a five residue insertion requires at least a seven residue fragment from the database[15]. While only few insertions in a family of homologous proteins are longer than 14 residues, there are many insertions that are longer than five residues[22]. Based on these studies in the mid and late 90ies much recent research shifted to full conformational search approaches, since those methods are not limited by the size of the available database but rather on our understanding of physics that guides folding of local conformations [5,9,22-27]

A recent analysis argued for a more favorable coverage of loop conformations in PDB. Du et al. [28] divided loop structures between a "template" and a "query" databases and compared these sets. They extrapolated that for seven residue fragments there was a 99% probability that a similar fragment is found within 0.7 Å RMSD, and even for long loops (15 residues) there was a high probability that there exists a non-homologous structure within 2 Å RMSD, considering Cα atoms only.

The number of sequences and structures dramatically increased over the last few years; accordingly, the difference in the sizes of sequence and structure databases is larger than ever before. Nevertheless, in part due to the efforts of Protein Structure Initiative, it will be possible to provide a reasonable model for most protein sequences within approximately 5 years, where more than 99% of these models will be generated by comparative modeling.

The practical questions are the following: is the current structural databank useful to supply fragments from various unrelated folds to complete these comparative models in their loop regions for any query that may emerge from the current sequence databanks? In case it is not, is there a promising trend towards this goal? In other words, are there many unique sequence and/or structural fragments being deposited to databanks?

In this work, we explore the question of what fraction of loops extracted from all known protein sequences (*Sequence Space*) is covered by loops extracted form all known protein structures (*Structure Space*). Our approach differs from the ones described in the past [11,21,28] because we do not restrict our investigation on assessing loop sampling on known protein structures but we estimate the current structural coverage of short segments in the Sequence Space, i.e. in the entire set of known sequences. Fragments from Structure Space were structurally clustered after an all-to-all comparison and sequence identity cutoffs assuring structural similarities were identified for each loop length. Next, all possible loop fragments from clusters of Sequence Space were matched with the sequences from Structure Space, and the coverage assessed. We also investigated the growth and change in the databases by repeating these exhaustive comparisons between sequence and structure databases that were available in 2001 and now. We focused our analysis on "medium" and "long" loops that are in the range of 8 to 14 residues.

## Results

### Structure Space

**Figure 1:**
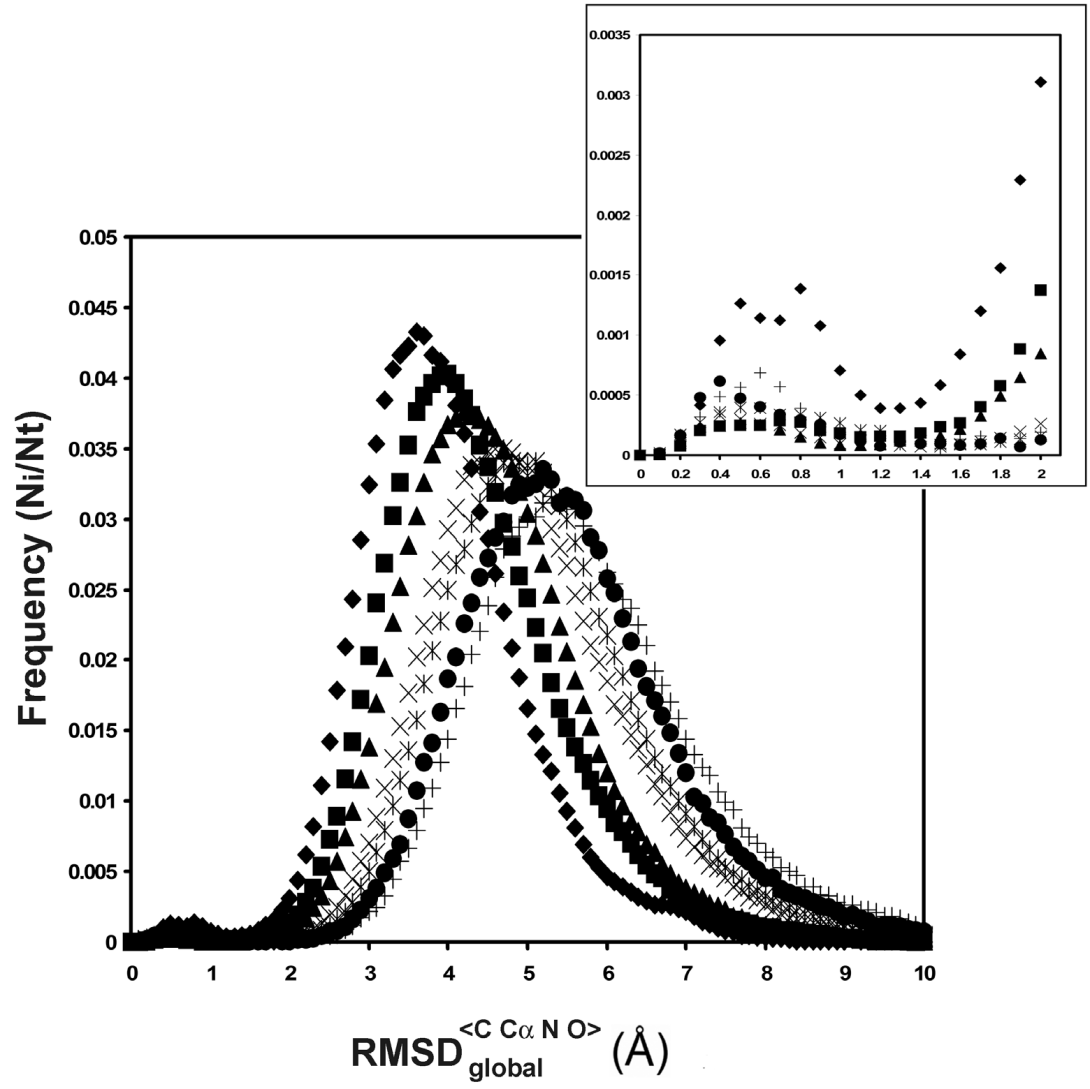
Histogram of RMSD_global _values after an all-to-all structural comparison of loops in *Structure Space*. The distribution of RMSD_global _values are grouped into bins of 0.1 Å. Loop lengths and symbols correspond as: 8 (◆), 9 (■), 10 (▲), 11 (× ), 12 (Ж), 13 (●), and 14 (+). Insert plot shows a blow up of the RMSD_global _distribution between 0 to 2 Å.

The Structure Space is composed of 105,950 loop segments with lengths between 4 and 14 residues. The histogram of distribution of the RMSD_global _values of an all-to-all structure comparison within each loop length class is shown in Figure 1. The distributions reflect the expected values from random comparisons and show bell-shaped curves with peak RMSD_global _values of 3.6, 3.9, 4.1, 4.7, 4.9, 5.1 and 5.4 Å for loop lengths of 8, 9, 10, 11, 12, 13 and 14, respectively. These are the expected accuracies if one assigns loop conformation to target segments by chance. At each length there is a small peak in the distribution in the range of 0 to 1.0 Å that refers to the small subset of related loops (inset in Figure 1). There is a clear transition between the RMSD distribution of related and unrelated loops at all lengths.

### Sequence identity as a function of structural similarity in the Structure Space

**Figure 2:**
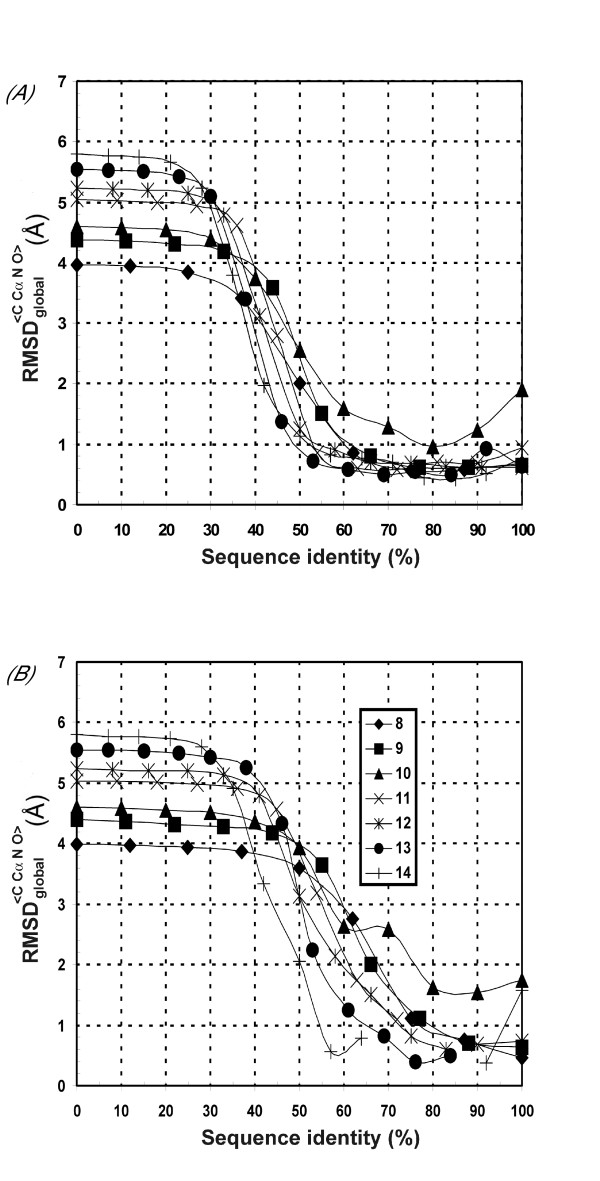
Average RMSD_global _(Y-axis) is computed as a function of percentage sequence identity (X-axis) for loops of 8 (◆), 9 (■), 10 (▲), 11 (×), 12 (Ж), 13 (●), and 14 (+) residues long, respectively, for a set of loops extracted from proteins (A) sharing less than 95% sequence identity (B) that belong to different SCOP families.

The shorter the sequences compared the higher sequence identity is required between them to confer structural similarity. The structural similarity (RMSD_global_) as a function of sequence identity is shown in Figure 2a. The standard deviations of averages (not shown) are around or below 1.5 Å in the range of 0 to 50% sequence identity and drops to around 0.5 Å when sequence identity increases above 50%, for all loop lengths of 8 to 14 residues. The decrease of RMSD_global _variation indicates that as sequence identity increases, consistently low values of RMSD_global _are obtained for pairs of superposed loops. In the range of 42 to 55% sequence identity there is a sharp transition between high to low RMSD_global _values at all lengths. When loops were filtered for redundancy by removing proteins on a SCOP superfamily or family level similar trends can be observed, however, with much smaller number of cases the observed. In other words, here we identify the set of loops, which are structurally very similar but belong to different structures. The sequence identity range of transition between high (>3 Å RMSD) and low (<2 Å RMSD) for these subsets of loops is shifted to the range of 50–64% (Figure 2b).

In general, a partially different trend is followed by loops of length 10. Instead of a monotonous decrease of RMSD_global _values with increasing sequence identity there is a spike in RMSD values among highly similar loops. Most of the 10 residues long loops close to or at 100% sequence identity level belong to the complementarity determining regions (CDRs) of immunoglobulins. These loops are involved in the recognition of the antigen molecules and have been shown that for five of the six CDR loops (also known as the hypervariable regions) there are few different main-chain conformations (canonical forms)[29]. The sixth loop is highly variable and it is involved in binding specificity. Out of total 590 loop pairs with 100% sequence identity, 489 pair show a RMSD_global _larger than 1.0 Å and all of these pairs are CDRs. A more general overview of this question is provided in Table 1: the number of loop pairs with identical sequence to one other with RMSD_global _values of 0–1, 1–2, 2–3 or more than 3 Å is listed. Apart from the unique but large subset of hypervariable CDR loops there is a strong correlation between conformational and sequence conservation at all lengths. Table 2 contains statistics on structural similarity about loops at the other extreme: pairs of loops with unrelated sequence (25% or less sequence identity) but with similar conformations. Although it is a relatively small fraction of all loops compared, but nevertheless hundreds or even a few thousands of pairs of loops exhibit a highly similar conformation (<1 Å) even though there is an apparent lack of sequence similarity.

Sequence identity cutoffs were identified for each loop lengths that guarantee conformational similarity (<1 Å). For instance, for loop length 8 a 62.5% cutoff is required to guarantee similar conformations but in case of 12 residue loops 41.6% cutoff is already enough to ensure structural similarity (Figure 2a and Table 3 and 4). These cutoffs values were used to infer structural relationship among loops using sequence information alone.

### Sequence Spaces

We extracted 1,308,121; 1,071,712; 884,632; 720,202; 586,760; 476,596; and 387,190 loop sequences for lengths 8, 9, 10, 11, 12, 13, and 14 respectively from 1,730,689 protein sequences that compose the Sequence Space *2005*. In case of Sequence Space 2001 we obtained 263,032; 213,973; 176,200; 141,832; 114,769; 92527; and 74613 loops for lengths 8, 9, 10, 11, 12, 13, and 14 respectively, from 350,412 protein sequences. This indicates an approximately 6 fold increase in the number of short fragments between years 2001 and 2005.

### Comparing Structure Space and Sequence Space 2005

**Figure 3:**
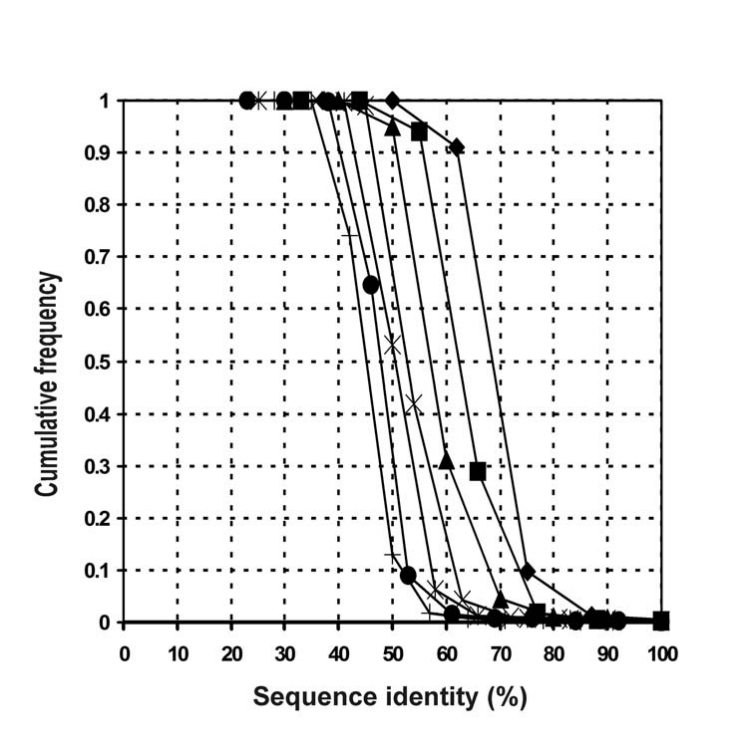
Cumulative frequency distribution of sequence identity comparing *Sequence Space 2005 *to *Structure Space 2005 *for loops of 8 (◆), 9 (■), 10 (▲), 11 (×), 12 (Ж), 13 (●), and 14 (+) residues long. The cumulative frequency at a given sequence identity value indicates the fraction of fragments in *Sequence Space *that has the corresponding or higher sequence identity to at least one loop in the *Structure Space*.

With the identified minimum sequence signal (Figure 2) that guarantees a structural similarity we can assess the fraction of loop conformations (as obtained from the Sequence Space) that is covered by known fragments in the Structure Space. Figure 3 shows the cumulative frequency distribution of percent of sequence identity of fragment pairs after an all-to-all sequence comparison of loops from both Structure Space and Sequence Space 2005. The frequency is accumulated from right to left, i.e. each value indicates the cumulative fraction of fragments in Sequence Space that share a corresponding or greater sequence identity to at least one loop in the Structure Space. Below 40% sequence identity only 20 and 10% of loops of length 13 and 14 from Sequence Space 2005 cannot be matched to at least one loop from Structure Space, while all other loop lengths matched at 100%. Meanwhile all loops (100%) of length 8 from Sequence Space 2005 have at least one loop in Structure Space at 50% or larger sequence identity. These percentages of coverage at 50% sequence cutoff drop to 96, 94, 68, 53, 33 and 13% for loops of length 9, 10, 11, 12, 13, and 14, respectively (Table 3). There is not a single loop in the current sequence space at any length up to 14 residues that is not matched with a conformational segment that shares at least 20% a sequence identity. Moreover, this minimum observed similarity is 40% for loops of 12 residues or shorter and is as high as 50% for 10 residue or shorter loops. As we recall an all-to-all structure comparisons (Figure 2a and Table 3) indicate that a 50% sequence identity will essentially ensure a structural similarity at any of these loop lengths

### Tracking trends in Sequence and Structure Space

**Figure 4:**
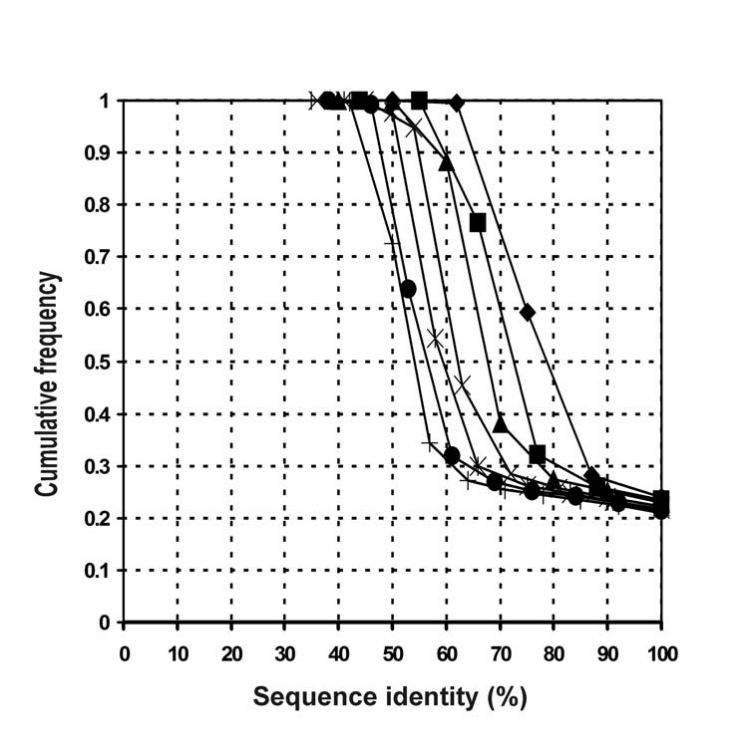
Cumulative frequency distribution of sequence identity comparing *Sequence Space 2005 *to *Sequence Space 2001 *for loops of 8 (◆), 9 (■), 10 (▲), 11 (×), 12 (Ж), 13 (●), and 14 (+) residues long. The cumulative frequency at a given sequence identity value indicates the fraction of fragments in *Sequence Space *2005 that has the corresponding or higher sequence identity to at least one loop in the *Sequence Space *2001.

We observed a strong coverage of structural loop fragments for the currently available sequence database. We made this comparison with the assumption that the current sequence database of approx 3 million protein entries is a good representation of all expected sequences. Next we explored two more issues: whether it is a correct assumption that the current, Sequence Space 2005 contains a near exhaustive compilation of all possible loop sequences. Second, if the good structural coverage of fragments observed is a consequence of a recent expansion of PDB or if it was the case earlier, in 2001 as well. Following the same approach described in the previous section, we compared Sequence Space 2005 against Sequence Space 2001. Figure 4 shows the cumulative frequency distribution of percentage of sequence identities for loops between 8 to 14 residues. One observes a 100% sequence identity for a large, 20 to 25% fraction of loop pairs at all explored lengths. A sharp transition takes place in the range of 40 to 75% sequence identity. At 50% sequence identity 100% of loops of length 8, 9 and 10, more than 95% of loops of length 11 and 12, 80% of loop of length 13 and 73% of length 14 are matched with at least one segment between the two Sequence Spaces (see also Table 4). This fraction indicates that while sequence databases keep growing at an exponential rate there are almost no unique conformational segment deposited up to 12 residues long fragments during the last 5 years.

**Figure 5:**
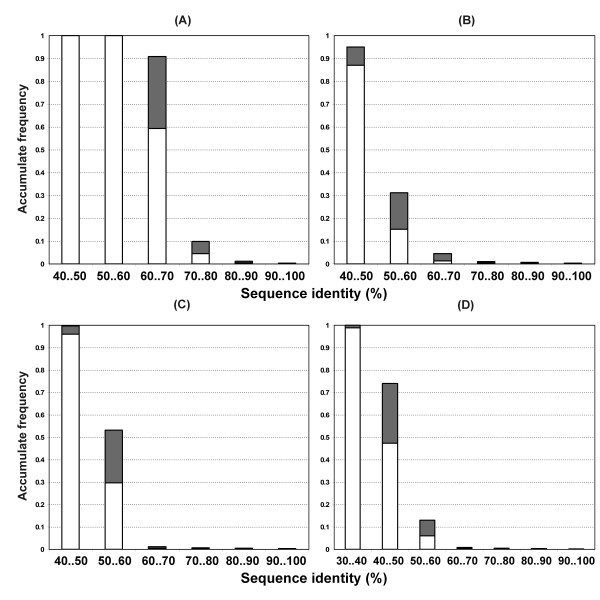
Comparing conformational fragment coverage in PDB in 2001 and 2005. Grey bars indicate the gain in structural coverage between 2001 and 2005 for loops (A) 8, (B) 10 (C) 12 and (D) 14, respectively.

The impact of the growing PDB was assessed by using a 2001 version of PDB to predict all possible sequences in the Sequence Space of 2005 (Figure 5). While there is an incremental improvement over the last five year period in coverage, especially at longer loop lengths, the availability of loop fragments was already good in 2001. While the ongoing saturation of conformational loop fragments will ensure an even finer granularity (delivering fragments at higher than 50% identity), essentially all possible query segments were already matched at 50% with a known structure in 2001.

### Impact of sequence identity in loop prediction

**Figure 6:**
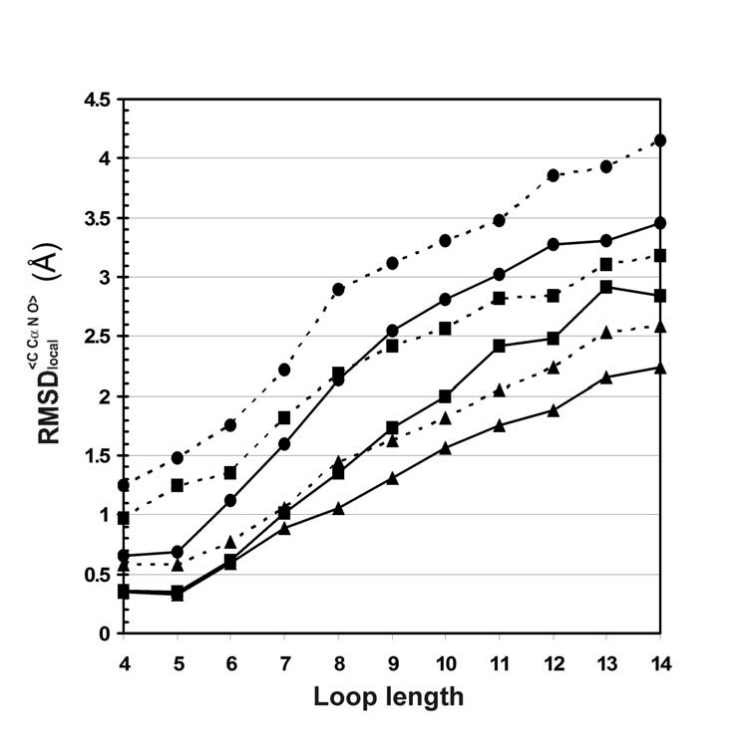
Accuracy of fragment search based loop prediction using sequence  information only (dashed lines) and using a prediction method[12]  (continuous lines) using 3 different databases, filtered for redundancy with regards to the query at 25% (▲), 50%(■), and 75%(●) sequence identity, respectively.

The structural similarity so far was exclusively related to sequence signal. This is a conservative approach as it was shown in Table 2: although it is a small fraction of the total number cases but at each length there are hundreds or even thousands of loops that are structurally very similar despite the apparent lack of sequence signal. If a prediction method locates these loops an even better coverage can be achieved than what was discussed above. Therefore we tried to estimate the fraction of loops that can be covered by known fragments if not only sequence information is used alone but some additional parameters. We compared two different scenarios: (1) Similar loop structures were predicted using sequence signal only; and (2) loops of similar conformation were predicted using a more elaborated prediction algorithm [12] that includes information about the geometrical fit of stem regions, 3 types of angles and a distance, the fit of preferred and observed main-chain dihedral angle preferences, and the fit of a template loop in a given protein environment with regards to steric clashes and non-boned contacts. In order to make both scenarios comparable under different conditions, we ran loop prediction by applying various sequence cut-offs (25, 50, 75%) to pre-filter the available structure database as compared to the query loops. This dynamic filtering approach was necessary because a prediction method using a structure database that offers sequentially obviously similar loops will not benefit much from a more sophisticated approach, and vice versa, a structure database that is overly cleaned up from trivial sequence similarities will be unfairly punishing sequence only methods, further, it would not be reflecting the observed good coverage in actual databases. The accuracies of predictions are shown in Figure 6. As sequence identity grows the prediction becomes more accurate but always the accuracy achieved is higher for the prediction algorithm than for the simple sequence identity lookup algorithm. Essentially the prediction method maintains the same accuracy as obtained for sequence only approach but for 1–3 residues longer loops as well. The longer the actual segments the bigger improvement can be achieved. Also, the gain in prediction accuracies increase as sequence identity of available segments drops, so other search factors weigh in.

## Discussion and conclusion

Short identical fragments can have completely different conformations [30,31]. However, these examples are rather rare exceptions to well established trends. As it is shown in Table 1 and 2 and in Figure 2a this is in general a highly unlikely situation. Sufficient sequence conservation -even for short segments- implicates structural similarity. This fact has been exploited in various database search dependent loop structure prediction methods. The shorter a given sequence the higher identity is required in general to assume structural similarity. For the medium and long loops (8–14 residues) studied here a 40–60% sequence identity was found to be a conservative limit to ensure structural similarity. Using a 40% sequence identity cutoff only about 5% of all studied short segments do not a match to a known fragment in the current structure space (Table 3 and Figure 3). Given the current numbers in PDB (Structure Space) and UniProt databases (Sequence Space 2005) there is a >95% coverage for loops up to 10 residues long. This favorable coverage is probably a consequence of the enormous expansion of PDB in the last few years, partially because of the Structural Genomics efforts that amalgamate the databank with new or remotely similar folds[6]. When sequence datasets were compared, we found that although the number of fragments in Sequence Space 2005 is almost 6 times larger than in Sequence Space 2001, this expansion is not reflected in form of many structurally new loop sequences. Almost all loop fragments in 2005 can be matched sequentially to a fragment already known in 2001 with 40% of greater sequence identity, and 25% of all loop sequences in 2005 have a 100% sequence identity to loop sequences in 2001 (Figure 4). This indicates that sampling of loop segments up to 12 residues in sequence databases might be close to saturation and a near full structural coverage is available for up to 10 residue long segments.

All our calculations were made and conclusions drawn in a conservative manner, using sequence information only as our sole assessment. It is safe to assume that the structural coverage of short segments is substantially larger than our estimations. We simulated a loop prediction exercise that includes not only selection and ranking of candidate loops by sequence information alone, but fitting the loops in the new protein environment and the assessment of their conformational fit. In this scenario the earlier established sequence identity thresholds can be less strict. As it is shown in Figure 6, for any given sequence identity threshold, the accuracy of loop prediction is always better. Essentially the prediction method extends the applicability of prediction (without loosing its performance) by approximately 2–3 virtual residues at each length.

According to the results reported here, the bottleneck in database search based loop modeling approaches is likely to shift from issues of database completeness of suitable fragments (sampling) to the development of novel scoring schemes that are capable of efficiently and accurately recognizing similar conformations. Once these techniques are available they seem to provide a dense coverage of loop segments for modeling studies.

## Methods

### Loop structure dataset

A representative set of 6578 protein structures were selected from the PDB [32]. The selected proteins were clustered and filtered so that any two share less than 95% sequence identity and all of them are determined by X-ray crystallography at a resolution of 2.5 Å or better. The DSSP program [33] was used to locate loop segments i.e. fragments that connect two regular secondary structures. The initial dataset of loops was further filtered by various quality rules: (i) loops with missing residues and/or main chain atoms (including Cβ, except for Gly), and (ii) loops with high crystallographic B-factors were discarded. The final set contains 105,950 protein loops of length ranging from 4 to 14 residues long. This compilation of loops will be referred as *Structure Space*.

### Loop sequence datasets

Two sequence datasets were considered using the sequence databanks from 2005 and 2001. Loop sequences were extracted from the 2005 release of UniProt catalog [34]. In order to have a comprehensive set of sequences but avoiding obvious redundancies the dataset UniRef90 v.6.0 containing 1,730,689 clusters or representative sequences was used. Secondary structures were predicted for all these sequences using PHD program [35], loops were defined as segments connecting two regular secondary elements. Only those α-helices and/or β-strands were considered that span at least 5 and 4 residues, respectively. We refer to this set as *Sequence Space 2005*. Loop sequences for dataset *Sequence Space 2001 *were compiled in a similar manner, except that instead of UniProt, that did not exist in 2001, protein sequences from the current SWISSPROT and TrEMBL databases [36] were downloaded. Those sequences that were deposited in 2001 or before were collected. The program CDHIT [37] was used to remove redundancy at 90% sequence identity that resulted in 350,412 clusters. This dataset is referred as *Sequence Space 2001*.

### Loop prediction datasets

Eleven test sets, each of which had 50 randomly selected loops from the Structure Space, between lengths 4 to 14 were used to test the performance of the prediction using only sequence signal or a loop prediction method. In order to explore different database conditions, several pre-filtered structure databases were prepared. Fragments were removed above a certain sequence identity cutoffs for each query segment using various cutoff values (25%, 50%, 75%).

### Assessing structural similarity between loops in *structure space*

The structure similarity was measured by the root mean square difference (RMSD) of the atomic coordinates. In order to fit a segment into the surrounding structure, an overlap at each end must be allowed. The optimal number of overlapping residues is three, two residues at the N-terminal and one residue at C-terminal of the segment[15]. Then, for a loop of length 8, 11 residues are considered, 2 in N-terminal, 8 in loop, and 1 in C-terminal regions. We referred to this RMSD values as the global RMSD (RMSD_global_). The main chain atoms (N, Cα, C and O) were used for RMSD calculation. Structure alignments and RMSD were calculated with the MODELLER package [38].

### Cross referencing Sequence and Structure Spaces

Loop sequences from Sequence Space were aligned against loop sequences form Structure Space and sequence identities were computed as the percentage of identical aligned position. The sequences from Sequence Space were aligned with the set of loops with the same length ± 2 residues from Structure Space and the highest sequence identity was kept (i.e. a loop of length 3 from sequence space was compared with loops of length 1, 2, 3, 4 and 5 of Structure Space adding as many residues from flanking secondary structures as needed). The wobbling of ± 2 residues was allowed in order to compensate for the errors produced by secondary structure prediction method when predicting secondary structure boundaries [39,40].

## Abbreviations

RMSD: Root Mean Square Deviation; Å: Angstrom; PDB: Protein Data Bank;

## Authors' contributions

NFF: design, acquisition, analysis and interpretation of data, drafting the manuscript. AF concept, design, analysis and interpretation of data, writing, finalizing manuscript.

## Figures and Tables

**Table 1:** Structural variation (percent distribution among RMSD bins) of loops with identical sequences.

**Loop length**	**Number of Pairs**	**0–1 Ang.**	**1–2 Ang.**	**2–3 Ang.**	**> 3 Ang.**
**8**	332	318	23	1	0
**9**	286	256	23	6	1
**10**	590	101	182	286	21
**11**	211	151	21	33	6
**12**	98	88	5	2	3
**13**	69	57	10	1	1
**14**	56	44	8	3	1

**Table 2:** Structural variation among sequentially unrelated loops (loops with less than 25% sequence identity.)

**Loop length**	**Number of Pairs**	**0–1 Ang.**	**1–2 Ang.**	**2–3 Ang.**	**> 3 Ang.**
**8**	1281596	5077	13867	215875	1046777
**9**	1231208	425	4927	114366	111490
**10**	1216131	342	2734	72567	1140488
**11**	1273594	108	935	35397	1237154
**12**	928413	408	915	17222	909868
**13**	527656	173	246	5603	521634
**14**	392981	245	232	3078	389426

**Table 3:** Observed fraction of loop fragments of various length (8–14 residues) that share at least the corresponding level (40%, 50%, 60%) of sequence identity with at least one other loop when comparing datasets of Sequence Space 2005 and Structure Space 2005.

**Loop length**	**Sequence identity**		
	
	**40**	**50**	**60**
**8**	100	100	92
**9**	100	96	65
**10**	100	94	30
**11**	100	68	18
**12**	100	53	5
**13**	90	33	2
**14**	80	13	1

**Table 4:** Observed fraction of loop fragments of various length (8–14 residues) that share at least the corresponding level (40%, 50%, 60%) of sequence identity with at least one other loop when comparing Sequence Space 2005 and Sequence Space 2001.

**Loop length**	**Sequence identity**		
	
	**40**	**50**	**60**
**8**	100	100	100
**9**	100	100	90
**10**	100	100	89
**11**	100	98	60
**12**	100	97	49
**13**	100	79	35
**14**	100	73	31
